# Supplementary material for: A novel combinatorial approach integrating experimental and computational analysis of antioxidant activity: Evaluating catechin and L-ascorbic acid in serum
Source: PLoS One. 2025 Jan 14;20(1):e0309881. doi: 10.1371/journal.pone.0309881 (PMC11731726; doi:10.1371/journal.pone.0309881)
Supplement: S1 File — (DOCX) [file pone.0309881.s001.docx]

**Tables S1** and **S2** list commercial serum samples that were purchased for this study from Discovery Life Sciences, Inc. (Los Osos, CA). As stated on their website, “For all clinical research activities, Discovery Life Sciences utilizes Institutional and independent Investigational Review Boards (IRBs) and Ethics Committees (ECs) that are in full compliance with Federal Regulations 21 CFR parts 50 and 56 and in accordance with regulations described in DHHS 45 CFR 46, 160 and 164, and the Health Insurance Portability and Accountability ACT of 1996 (HIPAA).”Table S3 lists the Cartesian coordinates of all the HAT complexes in the gas phase.

Table S1. Normal Triglyceride Serum Samples

| Product ID: | Triglyceride Result (mg/dL) | Age | Ethnicity | Gender |
| --- | --- | --- | --- | --- |
| DLS13-13764 | 57 | 65 | U/A | F |
| DLS13-13533 | 65 | 82 | U/A | F |
| DLS15-15647 | 70 | 62 | U/A | F |
| DLS16-75688 | 79 | 31 | U/A | M |
| DLS16-76131 | 85 | 63 | U/A | F |
| DLS16-06142 | 88 | 7 | U/A | M |
| DLS16-76264 | 90 | 72 | U/A | F |
| DLS16-76193 | 101 | 57 | U/A | M |
| DLS16-76390 | 115 | 29 | U/A | M |
| DLS13-13641 | 109 | 59 | U/A | F |
| DLS13-13477 | 119 | 55 | U/A | F |
| DLS13-13422 | 124 | 68 | U/A | F |
| DLS16-76231 | 130 | 60 | U/A | F |
| DLS16-75435 | 135 | 85 | U/A | M |
| DLS16-75198 | 144 | 68 | U/A | F |

*U/A means unavailable

Table S2. Severely Hypertriglyceridemic Serum Samples

| Product ID: | Triglyceride Result (mg/dL) | Age | Ethnicity | Gender |
| --- | --- | --- | --- | --- |
| DLS16-79764 | 817 | 39 | U/A | M |
| DLS16-75935 | 827 | 32 | U/A | M |
| DLS16-80285 | 829 | 34 | U/A | M |
| DLS16-79703 | 868 | 38 | U/A | F |
| DLS16-79834 | 921 | 34 | U/A | M |
| DLS16-82704 | 927 | 48 | U/A | M |
| DLS16-79721 | 939 | 43 | U/A | M |
| DLS15-25195 | 965 | 51 | U/A | F |
| DLS15-25703 | 975 | 39 | U/A | M |
| DLS16-80111 | 983 | 46 | U/A | M |
| DLS15-25793 | 994 | 54 | U/A | F |
| DLS16-75575 | 995 | 29 | U/A | M |
| DLS15-25099 | 1053 | 36 | U/A | M |
| DLS15-25438 | 1096 | 66 | U/A | M |

*U/A means unavailable

Table S3. Cartesian Coordinates of all the HAT complexes in the gas phase

Ascorbic Acid

C -0.432692 0.160444 -0.556188

O 0.042533 -1.177331 -0.453659

C 1.364443 -1.160884 -0.154573

C 1.823553 0.226503 -0.099733

C 0.782545 1.014851 -0.377700

O 2.035391 -2.139079 0.014586

O 3.098902 0.522277 0.170041

H 3.565500 -0.316620 0.294313

O 0.743760 2.356176 -0.398664

H -0.061024 2.666469 -0.826051

H -0.894445 0.285755 -1.546590

C -1.502670 0.428663 0.502050

H -1.850897 1.468145 0.347763

C -2.694011 -0.485762 0.324190

H -2.354306 -1.526532 0.413090

H -3.407678 -0.282147 1.134111

O -1.036509 0.229467 1.805994

H -0.288874 0.808093 1.982599

O -3.240726 -0.208738 -0.946568

H -3.902088 -0.868227 -1.161340

Zero-point correction= 0.150139 (Hartree/Particle)

Thermal correction to Energy= 0.162242

Thermal correction to Enthalpy= 0.163186

Thermal correction to Gibbs Free Energy= 0.111936

Sum of electronic and zero-point Energies= -684.461135

Sum of electronic and thermal Energies= -684.449031

Sum of electronic and thermal Enthalpies= -684.448087

Sum of electronic and thermal Free Energies= -684.499337

Ascorbic Acid 4OH

C -0.396094 0.195155 -0.578338

O 0.063317 -1.148221 -0.510278

C 1.382559 -1.217261 -0.153471

C 1.928014 0.203594 -0.063014

C 0.820823 1.021482 -0.392762

O 1.953759 -2.241255 0.014861

O 3.074623 0.509403 0.223026

O 0.845492 2.341897 -0.422822

H 0.007441 2.704535 -0.736464

H -0.878332 0.353984 -1.554885

C -1.441895 0.450397 0.516617

H -1.788833 1.494842 0.404029

C -2.637838 -0.457995 0.338579

H -2.299205 -1.499548 0.420422

H -3.346145 -0.256870 1.153684

O -0.932544 0.203578 1.796761

H -0.335650 0.905108 2.070708

O -3.187023 -0.169859 -0.927155

H -3.833687 -0.838630 -1.158302

Zero-point correction= 0.137082 (Hartree/Particle)

Thermal correction to Energy= 0.148875

Thermal correction to Enthalpy= 0.149819

Thermal correction to Gibbs Free Energy= 0.098227

Sum of electronic and zero-point Energies= -683.830943

Sum of electronic and thermal Energies= -683.819150

Sum of electronic and thermal Enthalpies= -683.818206

Sum of electronic and thermal Free Energies= -683.869798

Ascorbic Acid 5OH

C -0.440197 0.181896 -0.619712

O 0.007916 -1.160156 -0.340369

C 1.314496 -1.176221 -0.036125

C 1.838395 0.182308 -0.133682

C 0.782852 1.079300 -0.489856

O 1.955663 -2.148572 0.256923

O 3.091384 0.461619 0.117927

H 3.538460 -0.369823 0.357331

O 0.781383 2.288731 -0.605618

H -0.843688 0.196466 -1.637632

C -1.518707 0.595923 0.368464

H -1.893196 1.571778 0.023360

C -2.661172 -0.393932 0.401186

H -2.281982 -1.349919 0.788411

H -3.422944 -0.017980 1.098286

O -1.001070 0.671192 1.678547

H -0.665580 1.556526 1.839725

O -3.153666 -0.508845 -0.913690

H -3.777960 -1.234444 -0.960902

Zero-point correction= 0.138035 (Hartree/Particle)

Thermal correction to Energy= 0.149631

Thermal correction to Enthalpy= 0.150575

Thermal correction to Gibbs Free Energy= 0.099498

Sum of electronic and zero-point Energies= -683.849553

Sum of electronic and thermal Energies= -683.837957

Sum of electronic and thermal Enthalpies= -683.837013

Sum of electronic and thermal Free Energies= -683.888090

Ascorbic Acid 4OH-5OH

C -0.418157 0.282877 -0.654013

O 0.065490 -1.072936 -0.652574

C 1.328294 -1.200805 -0.182624

C 1.917075 0.204647 -0.024276

C 0.794987 1.159751 -0.434944

O 1.867964 -2.240535 -0.002187

O 3.016550 0.478720 0.335730

O 0.850618 2.348144 -0.525485

H -0.909069 0.474704 -1.612092

C -1.392137 0.487139 0.489481

H -1.741155 1.529967 0.418642

C -2.575672 -0.452855 0.410164

H -2.205951 -1.487448 0.420579

H -3.201638 -0.304887 1.305163

O -0.637321 0.262497 1.662322

H -1.087613 0.620437 2.430539

O -3.269310 -0.139455 -0.772444

H -3.932839 -0.808780 -0.948460

Zero-point correction= 0.125893 (Hartree/Particle)

Thermal correction to Energy= 0.137419

Thermal correction to Enthalpy= 0.138364

Thermal correction to Gibbs Free Energy= 0.087786

Sum of electronic and zero-point Energies= -683.256041

Sum of electronic and thermal Energies= -683.244515

Sum of electronic and thermal Enthalpies= -683.243570

Sum of electronic and thermal Free Energies= -683.294148

Catechin

C -1.799063 -0.390016 -0.075689

C -2.740255 -1.371480 -0.396830

C -4.093820 -1.068427 -0.398238

C -4.527397 0.206567 -0.086360

C -3.599258 1.199432 0.226138

C -2.251399 0.899290 0.227323

C -0.358370 -0.659366 -0.059588

C 0.261453 -1.839909 0.080380

C 1.739746 -2.007239 0.095746

C 2.436858 -0.688430 0.028360

C 1.721980 0.495261 -0.112615

H -2.418320 -2.372076 -0.650377

H -1.551566 1.690675 0.468237

H 2.036031 -2.551851 1.010573

O -5.837405 0.597660 -0.062025

H -6.410003 -0.132243 -0.309088

O 0.372505 0.510180 -0.190552

O -0.458857 -2.995759 0.190116

H 0.096212 -3.670505 0.590932

C 2.347250 1.733972 -0.190255

C 3.824369 -0.596393 0.092187

C 3.726809 1.788694 -0.122401

C 4.480230 0.624560 0.019770

O 4.306308 3.011892 -0.199991

H 5.261435 2.927213 -0.144966

O 4.495299 -1.770446 0.227561

H 5.440957 -1.605532 0.265291

H 5.566806 0.670183 0.072912

H 1.761464 2.638314 -0.302444

H 2.048018 -2.660722 -0.738062

O -4.009228 2.454197 0.528062

H -4.971690 2.481026 0.475582

H -4.823115 -1.835372 -0.651533

Zero-point correction= 0.246915 (Hartree/Particle)

Thermal correction to Energy= 0.265419

Thermal correction to Enthalpy= 0.266364

Thermal correction to Gibbs Free Energy= 0.200524

Sum of electronic and zero-point Energies= -1029.542743

Sum of electronic and thermal Energies= -1029.524238

Sum of electronic and thermal Enthalpies= -1029.523294

Sum of electronic and thermal Free Energies= -1029.589134

Catechin 3’OH

C 1.825574 0.307723 -0.046522

C 2.791472 1.300434 -0.368553

C 4.146635 1.010465 -0.379150

C 4.602710 -0.260715 -0.083897

C 3.657853 -1.332048 0.239200

C 2.262629 -0.965083 0.230256

C 0.391622 0.608267 -0.040453

C -0.202379 1.805814 0.080924

C -1.676898 2.001551 0.092845

C -2.400787 0.697469 0.026128

C -1.710648 -0.502884 -0.089024

H 2.464766 2.301446 -0.617406

H 1.565380 -1.762076 0.460852

H -1.962430 2.554986 1.005173

O 5.892720 -0.601127 -0.077155

O -0.358870 -0.547026 -0.144151

O 0.541136 2.943906 0.195145

H -0.030980 3.667906 0.463265

C -2.359755 -1.728407 -0.161613

C -3.790984 0.637139 0.070192

C -3.741529 -1.752302 -0.114263

C -4.471354 -0.570135 0.002173

O -4.344794 -2.962714 -0.186337

H -5.298931 -2.859657 -0.145931

O -4.435959 1.827560 0.181672

H -5.385953 1.686422 0.207719

H -5.559245 -0.591752 0.039035

H -1.792085 -2.646482 -0.253035

H -1.968055 2.658022 -0.744997

O 4.046668 -2.473869 0.502502

H 4.860590 1.792880 -0.630696

H 6.434785 0.160716 -0.306859

Zero-point correction= 0.233992 (Hartree/Particle)

Thermal correction to Energy= 0.252202

Thermal correction to Enthalpy= 0.253146

Thermal correction to Gibbs Free Energy= 0.186843

Sum of electronic and zero-point Energies= -1028.912391

Sum of electronic and thermal Energies= -1028.894180

Sum of electronic and thermal Enthalpies= -1028.893236

Sum of electronic and thermal Free Energies= -1028.959539

Catechin 4’OH

C -1.836792 -0.373218 -0.011016

C -2.813292 -1.408132 -0.168127

C -4.144693 -1.134120 -0.196030

C -4.631170 0.210596 -0.065265

C -3.612645 1.248775 0.087507

C -2.273847 0.960578 0.108341

C -0.419114 -0.646259 0.006936

C 0.216363 -1.846519 0.052685

C 1.695079 -2.002141 0.073822

C 2.395771 -0.687358 0.021822

C 1.678940 0.500496 -0.008239

H -2.481190 -2.430542 -0.274059

H -1.561934 1.767944 0.221729

H 1.983605 -2.564520 0.978987

O -5.822235 0.555131 -0.074400

O 0.325324 0.517689 -0.000143

O -0.484640 -3.003980 0.111464

H 0.121299 -3.748382 0.054271

O 2.300538 1.741187 -0.048739

C 3.785265 -0.603215 0.010106

C 3.682888 1.790648 -0.058795

C 4.439177 0.619547 -0.029815

O 4.258710 3.014858 -0.098580

H 5.215714 2.931821 -0.105168

O 4.452701 -1.785099 0.041677

H 5.401019 -1.630538 0.031526

H 5.526908 0.662044 -0.038142

H 1.713797 2.651343 -0.073449

H 2.002464 -2.641953 -0.772368

O -4.075601 2.488797 0.206104

H -5.048222 2.398657 0.158351

H -4.882350 -1.920222 -0.321801

Zero-point correction= 0.234659 (Hartree/Particle)

Thermal correction to Energy= 0.252625

Thermal correction to Enthalpy= 0.253569

Thermal correction to Gibbs Free Energy= 0.187135

Sum of electronic and zero-point Energies= -1028.932307

Sum of electronic and thermal Energies= -1028.914340

Sum of electronic and thermal Enthalpies= -1028.913396

Sum of electronic and thermal Free Energies= -1028.979830

Catechin 5OH

C 1.775078 0.387505 -0.076432

C 2.723711 1.359021 -0.405250

C 4.074348 1.043796 -0.409055

C 4.497548 -0.233446 -0.092017

C 3.561415 -1.216633 0.228793

C 2.216491 -0.904175 0.232579

C 0.338327 0.671404 -0.059295

C -0.273305 1.857525 0.085386

C -1.751667 2.041330 0.100661

C -2.451022 0.731784 0.033185

C -1.752969 -0.438404 -0.110324

H 2.410186 2.361413 -0.662373

H 1.512103 -1.688744 0.482353

H -2.056367 2.584255 1.012736

O 5.802671 -0.636639 -0.068985

H 6.383807 0.088144 -0.311586

O -0.407919 -0.493422 -0.191316

O 0.459526 2.999664 0.208698

H -0.107333 3.702710 0.538115

C -2.409754 -1.676353 -0.191373

C -3.905198 0.692083 0.109880

C -3.816601 -1.738752 -0.121834

C -4.555313 -0.599297 0.024550

O -4.344524 -2.979996 -0.210273

H -5.303536 -2.932939 -0.155393

O -4.551795 1.737483 0.242771

H -5.640181 -0.611910 0.082793

H -1.833163 -2.587817 -0.308845

H -2.067759 2.692858 -0.731669

O 3.960021 -2.472838 0.536553

H 4.922376 -2.509270 0.485756

H 4.809495 1.802966 -0.668205

Zero-point correction= 0.233556 (Hartree/Particle)

Thermal correction to Energy= 0.251793

Thermal correction to Enthalpy= 0.252737

Thermal correction to Gibbs Free Energy= 0.186452

Sum of electronic and zero-point Energies= -1028.916210

Sum of electronic and thermal Energies= -1028.897974

Sum of electronic and thermal Enthalpies= -1028.897030

Sum of electronic and thermal Free Energies= -1028.963315

Catechin 7OH

C -1.769153 -0.383755 -0.077510

C -2.689821 -1.379319 -0.412030

C -4.048599 -1.100898 -0.414174

C -4.505517 0.163121 -0.090884

C -3.596593 1.170185 0.235741

C -2.243668 0.894380 0.237618

C -0.324576 -0.628825 -0.060546

C 0.315483 -1.796438 0.088729

C 1.797059 -1.931475 0.096764

C 2.478160 -0.612835 0.024018

C 1.736517 0.571018 -0.125301

H -2.348811 -2.371131 -0.675451

H -1.559542 1.696142 0.489270

H 2.123078 -2.466834 1.007037

O -5.820430 0.530824 -0.066505

H -6.381742 -0.204719 -0.323136

O 0.387436 0.550379 -0.208810

O -0.379099 -2.966799 0.201592

H 0.154930 -3.608007 0.679389

C 2.354061 1.793795 -0.204912

C 3.893916 -0.529631 0.093610

C 3.794245 1.897637 -0.135147

C 4.541884 0.665600 0.019466

O 4.366002 2.993024 -0.204149

O 4.521062 -1.723568 0.235818

H 5.472400 -1.588290 0.275801

H 5.624528 0.741811 0.071776

H 1.775405 2.703003 -0.323469

H 2.126983 -2.577649 -0.735172

O -4.029051 2.412717 0.550089

H -4.991799 2.425393 0.495520

H -4.763168 -1.877677 -0.678506

Zero-point correction= 0.233538 (Hartree/Particle)

Thermal correction to Energy= 0.251704

Thermal correction to Enthalpy= 0.252648

Thermal correction to Gibbs Free Energy= 0.186493

Sum of electronic and zero-point Energies= -1028.913077

Sum of electronic and thermal Energies= -1028.894911

Sum of electronic and thermal Enthalpies= -1028.893966

Sum of electronic and thermal Free Energies= -1028.960121

Catechin 3’OH-4’OH-5OH-7OH

C 1.691938 -0.012464 0.096722

C 2.395550 -1.296090 -0.000184

C 3.724529 -1.362564 -0.139829

C 4.555447 -0.153426 -0.212558

C 3.818698 1.206111 -0.122790

C 2.358073 1.171299 0.032430

C 0.271590 -0.022501 0.277701

C -0.636237 -1.152544 0.281313

C -1.431674 -1.208024 1.595670

C -2.009952 -0.493196 0.433180

C -1.738096 0.922087 0.376868

H 1.796828 -2.200946 0.034469

H 1.846945 2.127208 0.093820

H -1.827903 -2.203697 1.769469

O 5.751631 -0.178155 -0.335345

O -0.351764 1.115234 0.474965

O -0.413366 -2.267354 -0.456029

H -1.310835 -2.604427 -0.694096

C -2.652516 1.835378 0.083685

C -3.212564 -1.017873 -0.303547

C -4.040629 1.347445 -0.269492

C -4.196575 -0.054298 -0.543556

O -4.918451 2.190248 -0.389276

O -3.179434 -2.223620 -0.613515

H -5.112573 -0.356430 -1.040646

H -2.464249 2.901720 0.026521

H -1.088080 -0.651280 2.461959

O 4.427565 2.244168 -0.175135

H 4.244928 -2.312356 -0.210486

Zero-point correction= 0.197015 (Hartree/Particle)

Thermal correction to Energy= 0.213743

Thermal correction to Enthalpy= 0.214687

Thermal correction to Gibbs Free Energy= 0.151609

Sum of electronic and zero-point Energies= -1027.026889

Sum of electronic and thermal Energies= -1027.010161

Sum of electronic and thermal Enthalpies= -1027.009217

Sum of electronic and thermal Free Energies= -1027.072295
